# COVID-19 vaccine attitudes among youth experiencing homelessness: a qualitative analysis with opportunities for action

**DOI:** 10.1186/s12889-023-16413-0

**Published:** 2023-08-31

**Authors:** Brandon Balma, Lauren Vasilakos, Ingie Osman, Asha Elgonda, Janna R. Gewirtz O’Brien

**Affiliations:** Department of General Pediatrics and Adolescent Health, University of Minnesota, Minneapolis, USA

**Keywords:** Youth, Homelessness, COVID-19 vaccine, Pandemic, Public health, Qualitative analysis, Adolescent health, Housing insecurity, Opportunity for action

## Abstract

Youth experiencing homelessness (YEH) are uniquely vulnerable to COVID-19 infection, yet are often excluded from response planning during the COVID-19 pandemic and other public health crises. As part of a larger community- and youth-engaged project through a national network of Prevention Research Centers, our qualitative study sought to describe youth perspectives that influence COVID-19 vaccine confidence and uptake, and identify youth-driven strategies to guide public health efforts to improve vaccine confidence and access. We conducted focus groups with youth experiencing homelessness (*n* = 20) and semi-structured interviews with staff members (*n* = 10) at youth-serving agencies to solicit youth perspectives about COVID-19 vaccination. Focus groups and interviews were recorded, transcribed, and analyzed using thematic qualitative analysis. In partnership with youth and cross-sector partners, we distilled eight salient themes that influenced COVID-19 vaccine uptake and confidence among YEH: 1. historical harms and mistrust of systems, 2. access to reliable health information, 3. prioritization of basic needs, 4. personal health influence, 5. barriers to healthcare, 6. fear and uncertainty of the vaccines, 7. sense of bodily autonomy, and 8. community influence. We also identified three youth-driven opportunities to increase COVID-19 vaccination among this population: emphasizing autonomy, leveraging trusted sources of information, and improving vaccine access.Our study elucidates perspectives of YEH on COVID-19 vaccination, and identifies several opportunities to improve youth vaccine confidence and access. It also underscores the importance of centering youth voice in response planning during current and future public health crises.

## Introduction

Youth experiencing homelessness (YEH) represent a diverse and remarkably resilient population of young people who face significant adversity, often as a result of multiple systems failures. Though definitions of youth homelessness vary by government agency and across the literature, herein, we focus on unaccompanied YEH, or those who are experiencing homelessness without a parent or guardian [1–3]. Likewise, we define the experience of homelessness broadly to include youth who have run away or been thrown out of their homes, are rough sleeping (sleeping in a place not meant for human habitation, in encampments, or “on the street”), staying in a shelter or housing program for youth, or couch-surfing [4, 5]. Importantly, youth frequently first experience homelessness as part of their family and subsequently experience homelessness on their own, highlighting the need for defining youth experiencing homelessness more broadly [6]. Estimates suggest that annually, there are 4.2 million YEH, ages 13 to 25, in the U.S. [3] and over 13,300 YEH in Minnesota [7], illuminating an extensive public health concern. Youth who are Black, Indigenous, and people of color (BIPOC) and 2-spirit, lesbian, gay, bisexual, transgender, and queer (2SLGBTQ +) represent a disproportionate number of YEH across the U.S. [1, 7, 8], again driven in large part by systems failures and marginalization [9].

The experience of homelessness represents a threat to the health and well-being of youth, which further compounds the health risks associated with several other intersecting and marginalized identities that many YEH hold. YEH have poorer physical and mental health outcomes when compared to their stably housed peers, thought to be related to a range of complex and interconnected factors, including structural racism, homophobia, transphobia, criminal justice systems involvement, trauma and victimization, limited educational opportunities, socioeconomic disadvantage, limited access to mental and physical healthcare, and the physical dangers associated with the experience of homelessness itself [10–14].

The COVID-19 pandemic has magnified these already stark inequities. Youth have been particularly vulnerable to entry into homelessness during the COVID-19 pandemic, related to many factors including financial strain, higher unemployment, loss of fundamental resources, and increased rates of domestic violence [14, 15]. Likewise, compared to stably housed individuals, people experiencing homelessness (PEH), are more likely to contract communicable illnesses and are more likely to experience adverse health outcomes related to vaccine-preventable illnesses, including COVID-19 [16, 17]. Before the COVID-19 pandemic, YEH were more likely to be under-vaccinated when compared to their stably housed peers [18], a trend that has persisted with COVID-19 vaccination rates among PEH [19–21]. Additionally, depending on the specific housing situation, following all guidelines for COVID-19 prevention is particularly difficult for YEH, such as hand-washing, mask-wearing, isolation, and quarantine, due to limited access to physical resources to implement these recommendations (e.g., inadequate running water and hand sanitizer, limited access to high-quality masks, lack of a stable home in which to isolate or quarantine). Additionally, YEH face poorer outcomes when infected with COVID-19 likely due to low vaccination rates, disrupted access to healthcare and other vital supports, and higher rates of underlying chronic health conditions [4, 22].

YEH are exceptionally resilient, demonstrate many strengths, and are the experts in their own experiences [23–25]. Yet, YEH are often excluded from conversations regarding their healthcare, social needs, and pandemic response planning. To describe youth perspectives on factors that influence COVID-19 vaccine confidence and access, we conducted a community- and youth-engaged qualitative analysis that identified youth-centered strategies to guide public health efforts to improve vaccine confidence and access.

## Methods

### Study design

We conducted a thematic analysis of qualitative data from focus groups with YEH and key informant interviews with staff at agencies serving YEH (herein, YEH-serving agencies) to describe YEH’s perspectives on COVID-19 vaccination, and identify youth-driven strategies for how to most effectively improve COVID-19 vaccine confidence and access among YEH. These data were collected as part of a community- and youth-engaged project, funded by the Centers for Disease Control and Prevention (CDC), focused on improving COVID-19 vaccine confidence through a national network of Prevention Research Centers. The project necessitated cross-sector collaboration between governmental public health, healthcare institutions, YEH-serving agencies, and youth with lived experiences of homelessness. To do this, we developed and fostered partnerships over the course of 14 months through monthly meetings to engage experts across youth-serving sectors in and around Hennepin County, Minnesota. Our cross-sector team included 33 individuals from 11 organizations from across sectors, including YEH-serving agencies, healthcare, and public health. Together, we agreed upon shared goals of identifying and implementing anti-oppressive, trauma-informed, youth-driven strategies to support YEH during the COVID-19 pandemic and beyond.

### Recruitment and data collection

Youth, ages 12–24, who were currently experiencing homelessness (defined as above) were recruited and invited to participate in focus groups through four local YEH-serving agencies across Hennepin County, MN, leveraging existing agency youth-boards and connections with youth that they serve. We conducted four, 60-min youth focus groups with a total of 20 youth (*n* = 20). Ten youth completed an optional demographic survey (six male, three female, one transgender/genderqueer) and indicated their racial identity (four White/Caucasian, two African American, one Hispanic/Latino, two Mixed Race). Ten similarly structured, 60-min key informant interviews (*n* = 10) were conducted with current staff members working at the YEH-serving agencies. Key topics covered regarding YEH included overall factors that influence COVID-19 vaccination, barriers to vaccination, ideas about strategies to obtain reliable vaccine information, and feedback about pre-existing vaccine messaging. Interviews and focus group sessions were audio-recorded, stored in a secure online platform, auto-transcribed, and subsequently double-checked and de-identified. All youth and staff participants were provided with $50 gift card incentives at the end of focus groups and interviews. Informed consent was obtained from youth and staff prior to participation.

This process reinforced privacy and confidentiality. We emphasized that participation was entirely optional, that they could choose to discontinue at any point, and that their participation would not influence any resources or healthcare they received in shelter. Interviews concentrated on inviting youth to share perspectives they heard from their peers rather than their own personal information in order to respect and protect their privacy. Focus group facilitators had extensive experience working with historically marginalized youth, as well as a robust knowledge of research ethics, privacy and trauma-informed care. If personal or sensitive topics arose, these facilitators were well prepared with resources to offer youth if needed. The University of Minnesota IRB determined that this study was not research involving human subjects; therefore, further IRB review and approval was not required.

### Analysis

We used Braun and Clarke’s method [26] of thematic analysis to identify YEH perspectives around COVID-19 vaccination from youth focus groups and YEH-serving staff interviews. To begin this process, two independent coders who were not involved directly in data collection, one undergraduate (BB) and one medical student (LV), reviewed all youth transcripts and noted initial emerging themes from the youth focus group transcripts. They each independently used an open-coding, summative approach to develop a high-level coding framework consisting of codes and subcodes. Both coders independently applied the codes to the four youth transcripts, using a deductive approach. To better establish reliability, the coders iteratively came together on a biweekly basis throughout the coding process to reconcile the entirety of each individual transcript and identify discrepancies in coding. Ultimately, agreements were reached on each coded excerpt and identically coded transcripts were established after repeated, thoughtful reconciliation. Updates to the codebook were made as needed to further refine codes throughout the analysis process. A miscellaneous category was created for excerpts that were felt to be relevant but did not clearly fit into the established codes. The miscellaneous category was then reviewed after the analysis to identify any additional key themes that emerged.

To keep the analysis centered on youth, we applied the youth-oriented codebook to the staff transcripts. Again, the same two coders (BB and LV) independently coded the ten staff transcripts and iteratively came together to reconcile each transcript. As distinct themes emerged from the staff interviews that did not appropriately fit into the already established codes, the codebook was updated to reflect these additional staff-derived perspectives and previously coded transcripts were reviewed for these additional themes. Two senior members of the team who were directly involved in data collection (JGO and IO) provided ongoing support throughout the entire analysis, which included attending initial coding meetings, reviewing all four youth transcripts, contributing to developing the initial coding framework, and helping reconcile any coding discrepancies. Their experience with data collection and familiarity with the study population helped provide context in developing the initial coding framework.

Following both youth and staff transcript coding, we generated a table from the codebook with representative quotes pulled from both the youth and staff transcripts to correspond with each code and subcode. Data were organized into two main categories, vaccine decision influencers and opportunities to promote vaccine confidence and access (Table 1 and 2). We used an iterative, community-engaged, reduction process to identify the most salient themes, note relationships between key themes, and to develop and then modify visual depictions of the data. Tables 1 and 2 depict the final coding framework. To do this, we generated visual depictions of the findings and presented these to the cross-sector team; the team provided input on intersecting, related, and salient themes.

**Table 1:** Codebook of vaccine decision influencers for YEH identified by youth and YEH-serving staff

Code	Definition	Sample Youth Quote	Sample Staff Quote
Access to reliable health information^a^	This code includes any references to formal and informal sources of information/misinformation regarding vaccines, as well as comments related to health education	“For a lot of the youth, they don’t really know nothing about the vaccine and half of the youth, they don't get nobody to explain and things and they not about to look it up for themselves. They just want to take the shot without knowing information and the people that is giving it to them, not going to explain to them. So, that's why it's caused a lot of problems with teenagers and with kids.”	“I mean I think there's so much distrust and not knowing what to believe. I think the misinformation and like just so many different, “Yes, it’s good, no it's not,” the back and forth, I think, that has impact. I think that's why, I personally think that’s why a lot of people don't get vaccinated or haven’t.”
Basic needs as youth’s priority^a^	This code includes multiple named basic needs that the youth prioritize and, therefore, take precedence over receiving the COVID-19 vaccine. For example, this includes named social barriers such as: transportation, childcare, employment, access to technology, and housing status	**Childcare:** “I would also say childcare. Cause I've heard that if you’re going to go get the COVID vaccine, you're technically not supposed to have more than one person back there with you.” **Employment and school time conflicts:** “When I was working, I had two jobs and going to school, so I didn't even have time for family. So, I'm missing birthdays and everything.” “Not being able to take time off work to get vaccinated, as being something that's a barrier.” **Hassle factor:** “I would also say weather conditions. It’s starting to get cold, rain.” **Housing status:** “Most homeless youth shelters probably want you to get the vaccine or either get tested every week or every two weeks, I think. So, by them not really having a choice of living, the agenda would be pushed more on them since, you know, it's more of a public space than inside your own home.” **Interpersonal conflict:** “Or for people who may be fully against having the vaccine and they know you have gotten it, they may just not want you around.” **Technology:** “Not having a phone to actually make the appointment.” **Transportation:** “Transportation. Number one. Not just for me. That’s one of my bigger ones, but that’s huge for a lot of people, too.”	**Childcare:** “Because we have a lot of youth who are parenting, so their main concerns are childcare, education, dental, and basic health care first, as opposed to I think I need to get a shot for something that a lot of people my age seem to be bouncing back from.” **Employment and school time conflicts:** “Some young people actually, who have jobs or who are trying to maintain their job, would say… ‘I decided to get the vaccine because of that, so that I can keep my job and protect myself, as well, and the population that we're serving.’” **Hassle factor:** “Knowing the process, I think, absolutely, knowing where to go, knowing the process, how to register, how to sign up your name, you know like, do you do have to go through the lines, do I have to take transportation, like how many buses do I have to take for this, do I have to get bus tokens for this?” **Housing status:** “I had some people tell me that because they didn’t have an address, they couldn't.” **Interpersonal conflict:** “But there are a few adults in their life who are telling them, don't trust it, don't listen. So I think they get caught in the whirlwind and the youth are the ones who suffer. They’re in the middle.” **Technology:** “A lot of our youth have very scattered access to phones. Very few of them have computers unless they’re in a school. So joining a zoom meeting to get information or access information can definitely be challenging.” **Transportation:** “I mean just in general, transportation. Almost all of our youth use the buses, so that definitely can be a challenge. And just knowing who is offering what, where, and when. Keeping track of all of that.”
Barriers to healthcare	This code includes any reference to accessing healthcare, including insurance, cost of vaccination, or personal documentation	“For me, my biggest influencer is insurance right now.”	“A lot of them don't have a regular doctor that they trust, they might not have health care”
Community influence	This code includes comments about community attitudes and relationships that impact vaccine decision making, such as youth wanting to protect their family, loved ones, and community	“I would say sick loved ones around you. So, say, for some people, their job is being the PCA nurse and to prevent possible things from happening to the person they’re watching, they may get the vaccine.”	“…they may be doing it because their school requires it, or because they want to be in the sport or, you know, some do it for family members…”
Fear or uncertainty of vaccine	This code includes any worry or uncertainty about receiving the vaccine, which includes the fear of vaccine side effects, vaccine composition/development, and needles	**Fear of side effects:** “What’s the long-term effects that it’s gonna have on my body futuristics?” **Fear of vaccine composition/development:** And why are there mRNAs in there, as well? Which is a very big concern why you have modified DNA ingredients in there. Very, very big concern **Fear of needles:** “I feel like another thing that could make it hard for a person to want to get vaccinated, if they have a fear of needles, then it might be hard for them to want to go in and take a shot because they don’t like to be poked with a needle.”	**Fear of side effects:** “I think the first thing they'll probably say is, “What about the long-term side effects? Because we still don’t know that.” You know, because that's usually been one of the biggest things that have come up, you know, in conversation is long-term side effects.” **Fear of vaccine composition/development:** “Maybe kinda also talk about what exactly are they putting in your arm, you know, what is the vaccine, what does it consist of. And, you know, because a lot of folks have also mentioned to me before, actually, in the past that they’re putting the virus in you…”
Historical harms and mistrust of systems^a^	This code includes any mention of rightful mistrust of systems. For example, any mention of mistrust of vaccine/testing mandates, systemic racism, government, structural harms, racial disparities, authority, or control	**Mistrust of healthcare system:** “At first, they said you couldn’t get it, but now they’re forcing you to get it. They said they wasn’t going to force y’all to get it. It’s basically a control thing, that’s what I’m saying.” **Mistrust of government:** “The government is using it as another way to get money.”	**Mistrust of healthcare system:** “If we take into consideration how communities are feeling, why they feel that way, but then also have some sense of history. I don't think it's just a Tuskegee experiment that many African Americans are holding onto as a reason on why we are hesitant about the vaccine. I mean as a history of Black women being forced to be sterilized in prisons in the United States. I could run down the gamut of medical mispractices with the African American community. But certainly, I think it's important to keep in mind how those people are feeling who have been the victims, or have been the guinea pigs of Big Pharma. I think that's important.” **Mistrust of government:** “But it's such a huge ask to ask people to trust the system that's harmed them before to give them a vaccination”
Personal health influence^a^	This code includes comments about the impact of youth’s perception of personal health on their decision to get vaccinated, for example how pre-existing health conditions or allergies impact their comfort in receiving the vaccine	“A reason they might not get it cause they already had experience with catching COVID and feeling like taking the shot might make it worse.”	“And there's the kind of dual narrative out there of either, you know, if they get it, they'll be fine or they're not going to get it because they're young, so it's not going to affect them as much. So, there's that front, as well, that we're fighting.”
Sense of bodily autonomy	This code includes any mention of the desire for having personal choice over one’s body, as well as concerns regarding the sense of losing bodily autonomy regarding healthcare and vaccination	They was telling them that it’s the test, but they’re actually giving it to them. But I– I was in jail and they told us to take the nose swab or whatever, but then they never told us our results. They never told nobody	Just to continue having an open door policy and make it available, and people to get to it on their own volition

Key themes, or codes, identified by qualitative analysis of youth and YEH-serving staff transcripts reflecting factors influencing COVID-19 vaccine confidence and uptake among youth experiencing homelessness (YEH). Of note, ^a^indicates the most salient themes identified by youth and cross-sector partners

**Table 2:** Codebook highlighting youth-driven opportunities for improved vaccine confidence and uptake among YEH

Code	Definition	Sample Youth Quote
Promoting autonomy	Encouraging the ability for youth to choose if/when/where/which vaccine to receive	“With the whole job thing and COVID-19, I get why they want people to have the vaccine, but I also get why people shouldn’t, if you know what I mean. I just think it would be helpful for people to have a choice instead of it being forced upon other people. Is that okay? I’m just stating my opinion.”
Utilizing trusted sources of information	Utilizing and creating youth-centered messaging and trusted youth spaces, disseminating information from vaccinated folks or immunologists, and ensuring that the information sources are factual and youth-friendly	“A dream solution being, like, a Tik Tok campaign, or other popular social media site, to get out correct information with sources, reputable sources.” “Another totally separate one I just thought of is specifically, like, hearing about what having COVID is like and side effects from COVID, rather than from the vaccine. And hearing that having COVID is worse than anything the vaccine can cause, then okay, that’s going to make me want to get the vaccine.”
Improving vaccine access	Developing improved access to vaccination, including opportunities that don’t require proof of insurance or personal documentation, providing transportation to vaccine sites, meeting youth where they’re at, and offering post-vaccination aftercare	“Taking good measures to make sure that these youth have proper resources and information to make an informed decision about it. And then offering them resources in order to find a vaccine.”

Key themes, or codes, identified by qualitative analysis of youth and YEH-serving staff transcripts identifying youth-driven opportunities to improve COVID-19 vaccine messaging and, therefore, vaccine confidence and uptake among youth experiencing homelessness (YEH)

## Results

### Factors influencing COVID-19 vaccine confidence and access among youth experiencing homelessness

**Fig. 1:**
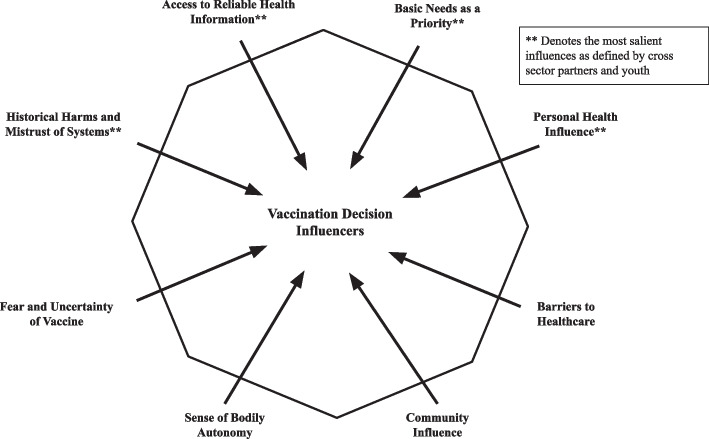
Vaccination Decision Influencers Identified by YEH and YEH-Serving Staff. Eight key themes were identified by youth and YEH-serving staff as factors influencing COVID-19 vaccine confidence and uptake among youth experiencing homelessness (YEH). Factors denoted with an asterisk were agreed upon by cross-sector partners and youth as being most salient for youth

Our analysis revealed eight factors that influenced COVID-19 vaccine confidence and access among YEH: 1. historical harms and mistrust of systems, 2. access to reliable health information, 3. basic needs as youth’s priority, 4. personal health influence, 5. barriers to healthcare, 6. fear and uncertainty of the vaccines, 7. sense of bodily autonomy, and 8. community influence. The former four factors were identified as most salient by youth and cross-sector partners. Figure 1 illustrates these eight themes and how they may influence YEH’s decisions to receive the COVID-19 vaccine.

#### Historical harms and mistrust of systems

Both YEH and agency staff articulated the influence of historical harms and the experience of marginalization and systemic oppression on their confidence in the COVID-19 vaccine. Mistrust of systems was particularly salient and emerged as an influence that likely impacted several of the other factors. Though we initially coded “historical harms” and “mistrust of systems” separately, the two codes were found to be intersecting and inter-related, and, based on input from our cross-sector community partners during the reduction process, we merged and re-coded as a single code. In interviews and focus groups, historical harms and marginalization by systems were demonstrated through elements of mistrust and uncertainty of healthcare delivery among youth, both within and outside of the context of the COVID-19 pandemic.

Both youth and staff frequently commented on the heavy impact that mistrust of the healthcare system had on their perceptions of the COVID-19 vaccine, resulting in rightful hesitancy to interact with healthcare, particularly as a person of color. For instance, one youth *said, “They’re lying. You know, some people think it's a population control attempt. You know, racist. Like, some certain drugs were made by the government to get rid of Black people. It happened.”*

Staff, who spend a lot of time conversing with youth at their agencies note that the government’s role in vaccine communication is complex. A staff member indicated government mistrust as a multi-faceted influence for youth, describing, *“There's definitely distrust because it's a systematically racist government, but I think also, they don't trust it because it's a government and they don't have that rapport with it. So, I think it's a combination of a two-sided coin and it's on both sides.”* Some staff members commented on the importance of recognizing healthcare’s historical harms in the context of vaccine discussions: *“Even in the medical field, there’s been very unethical things that have happened in the past, and just acknowledging that and apologizing for that, but saying this is really important for our safety. And this is what it looks like.”*

Several youth noted that the vaccine incentive system felt coercive and often augmented their skepticism about the vaccine:

- “Why are there incentives on it?”

- “Sorry, but if you give me money for something, that makes me skeptical on wanting to get it.”

- “I mean, if you're paying me, is there something wrong with it?”

- “Why are they paying us to get the vaccine?”

#### Access to reliable health information

Young people were often stretched in many ways as they consumed opposing information, which impacted their perspectives on the COVID-19 vaccine. Youth noted that their information sources included word of mouth, anecdotal stories, social media, other online platforms, school, information in public spaces, and information from trusted people in their life. Many youth reported confusion about not knowing which information to trust, particularly as the COVID-19 pandemic continued to evolve. For example, one youth noted, *“I don’t know, some people believe that the vaccine could be the virus, some people believe the vaccine could help you and protect you from it*.”

As one youth noted, misinformation and lack of reputable sources was a problematic influence for young people: *“Unless they cite their sources, unfortunately. It’s just– people can say whatever they want and people believe them, unfortunately. And it's a problem*.” Many staff echoed concerns regarding the inconsistent information being circulated to YEH. A staff member reflected on having a conversation with a young person who worried the vaccine would make her infertile: *“They’re not actually pushing facts, they’re pushing fear tactics and so let's talk about what that really means. And now she knows it's not the truth, but where you're getting your information from really matters and just having that conversation, really matters.”* The information that youth gathered about the COVID-19 vaccine, coupled with their lived experiences, influenced their decision on whether or not to receive the vaccine.

Youth shared that they received their information from many sources, which we classified as formal and informal. Formal sources were sources that have been classically used to convey health information, including physicians and nurses, parents/family, YEH-serving agencies, libraries, and online or phone-line nurses and clinicians. One youth states, *“My family is personally against it, being a Latina female. So, they wouldn’t take it.”* Informal sources of health information were sources that youth specifically reported were accessible during pandemic, including anecdotal stories from peers, social media, and celebrity opinions through social media. One staff member said, “*A lot of anecdotal trusted people in their lives. Anecdotal stories from a lot of people that are trusted people in their lives. I feel like it has led to a lot of people not getting vaccinated.”* Misinformation contributed significantly to the attitudes towards health information for YEH. For example, staff commented on the videos youth would view from TikTok and other social media that disseminated false vaccination information: “*The streams of information that is out there, whether it’s on social media, whether it’s in the social sphere, also plays a major influence and a decision they made, whether to or not get the vaccine.”*

#### Basic needs as youth’s priority

Both youth and staff discussed that YEH prioritized basic needs, which often took precedence over receiving the vaccine or seeking information about COVID-19. These unmet basic needs included access to technology, transportation, childcare, and housing (Table 1). Other named conflicts that inhibited youth from accessing basic needs included employment and school time conflicts, interpersonal conflicts, and the hassle factor of planning and following through with vaccination.

While many youth indicated prioritizing basic needs over receiving healthcare or the vaccine, some youth mentioned that getting the vaccine, along with monetary incentives, helped them obtain basic needs. One youth shared, *“It’s just scary. You know, I'm homeless, hungry. So, of course I did to get some money in my pocket, get some food in my stomach*.” Interestingly, some youth also commented about monetary incentives feeling coercive at times, which could have augmented mistrust of the vaccine (see Historical Harms and Mistrust of Systems above).

YEH’s focus on accessing basic needs was a salient consideration when staff reflected on low youth uptake of the COVID-19 vaccine: *“And also understanding that for homeless youth, this is not a priority for them. You know, they’re figuring out where they’re going to live, where they’re going to stay, what they’re going to eat.”* Additionally, one staff member commented on several barriers to basic needs that were intensified during the COVID-19 pandemic, which impacted where YEH focused their priorities: “*All the barriers still exist… people are experiencing super high rates of mental health issues, not being able to work, poverty, obviously, food insecurity.*”

#### Personal health influence

Several youth commented about how their personal health impacted their decisions to get vaccinated. Pre-existing health conditions, history of allergies, and former vaccine-related side effects were some examples youth reported that impacted their uptake of the vaccine. Concerns about their personal health resulted in worries about receiving the vaccine: *“I’ve reacted to just about every other vaccine of other sorts that I’ve taken, so, pretty afraid to take this one.”* Conversely, some youth indicated an interest in vaccination as a way to protect their personal health: *“A reason to take the vaccine, like, to ensure safety or just wanna make sure that they’re okay.”* Staff indicated that many youth felt that the consequences of COVID-19 infection were not relevant to them, as they considered themselves young and generally healthy: “*I would have to go back to how much they are aware of the severity of COVID. I think the general perception is that they're young and they'll be fine.”*

#### Barriers to healthcare

Youth indicated barriers to accessing healthcare, such as the belief that the vaccine process requires insurance, costs money, or requests personal documentation, such as citizenship documentation or proof of address, which influenced their decisions about COVID-19 vaccination. Concurrently, for YEH, obtaining and maintaining identification may be a barrier in itself, contributing to perceptions of what health and social services they may and may not be able to access. Unclear messaging about what was required was noted as confusing and stressful for some youth considering the vaccine: *“For me, it was specifically insurance. Because they’re like, ‘It’s free with most insurance!’ So that means you have to have your insurance all sorted out and have the right one.”* Conversely, youth also reported that if they were certain that the vaccine would be free to them and not require any form of insurance, identification, or documentation, then this would positively influence their decision about vaccination. Staff even noted the importance of youth having a trusted primary care provider that youth can rely on for trusted vaccine information and administration: *“A lot of them don't have a regular doctor that they trust.”*

#### Fear or uncertainty of vaccine

Several youth commented on their fears or uncertainties specifically related to receiving the COVID-19 vaccine, which influenced their confidence and uptake. Youth frequently reported fear of vaccine side effects, fear of vaccine composition and development, and fear of needles. Youth reported significant worries about both the long-term and short-term side effects from the COVID-19 vaccine, some of which was misinformation specific to the COVID-19 vaccine that they heard through various channels:

*“I have seen many different crazy side effects: deformities, people coming out and not being able to speak, some people coming out being paralyzed, all different side effects that's unique to different people… And just that kind of uncertainty makes it even less likely for me to even think about getting it.”*

Staff often remembered youth worrying about vaccine side effects. Some staff mentioned that youth have voiced concerns about fertility-related vaccine side effects, noting: *“That’s something that I think some youth have brought up. They worry about fertility.”*

Several youth also noted that their fear of needles influenced their decision about COVID-19 vaccination. One youth commented, *“That's the main part where the needle bothers me because it’s going into a muscle. Like that’s, ouch, because of the longer-lasting pain with that needle versus other needles.”* Fear, particularly in the context of youth-directed vaccine messaging, was also highlighted. For example, one staff member explained, *“So a lot of the concerns, paranoia, the aversion to the vaccine is stimulated not so much by reasoning, but I would say, it's on an emotional level. It's fear.”*

#### Sense of bodily autonomy

Many youth spoke about the importance of bodily autonomy when making their own decisions about COVID-19 vaccination. Youth expressed desire to be in control of what happens to their own body. When discussing how COVID-19 testing and vaccination specifically affect young people in shelters, one youth reported feeling exploited: *“They’re lab rats, to be honest.”* Another youth similarly indicated how being in shelter can specifically affect their sense of autonomy: *“Most homeless youth shelters probably want you to get the vaccine or either get tested every week or every two weeks, I think. So, by them not really having a choice of living, the agenda would be pushed more on them since, you know, it's more of a public space than inside your own home.”* The staff were similarly mindful of young people’s desire for bodily autonomy: *“If you feel like you’re being really pushed or coaxed into something, you have that reaction to push back. Just to say, it’s okay to have hesitancy. It’s okay to really think through this, and honor and give space for that.”*

#### Community influence

Young people’s relationships with others, both on an individual and community level, was a factor for many youth when deciding to get vaccinated. Protecting their loved ones or protecting their community by getting vaccinated was important to several youth: *“People might want to be vaccinated to protect people around them or their loved ones. People might want to be vaccinated to ensure their safety, make sure that they're okay.”* Community was also noted to be incredibly important for YEH, which includes protecting their community by limiting the spread of COVID-19 through vaccination, as well as youth being able to attend school or community recreational events through proof of vaccination. Staff also report this multi-factorial element of community influence: *“I've had a good number of young people who were in full support of getting vaccinated… as a way to protect themselves and their loved ones and one step to returning to a ‘new normal,’ if you may, just to be able to do things that they once enjoyed.”*

### Opportunities to promote COVID-19 vaccination among youth experiencing homelessness

**Fig. 2:**
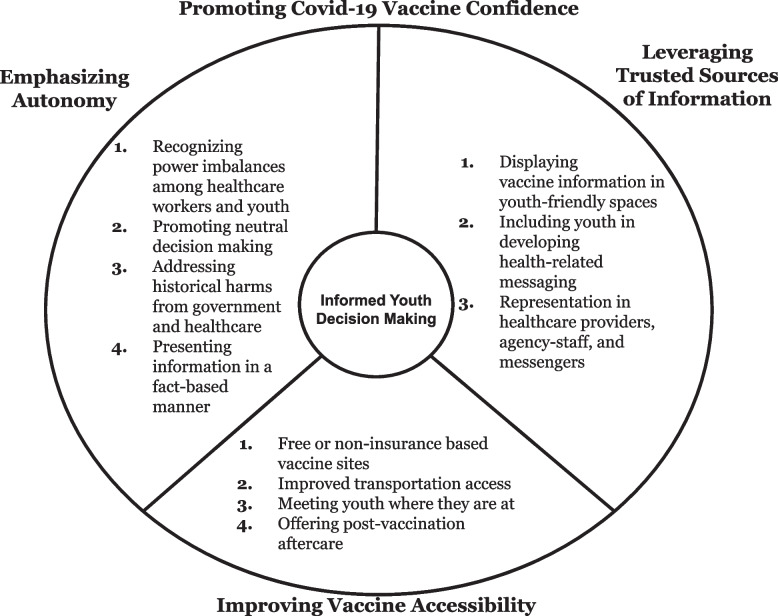
Promoting Covid-19 Vaccine Confidence among Youth Experiencing Homelessness. Three prominent opportunities were identified by youth as strategies to improve youth-centered COVID-19 vaccine messaging for youth experiencing homelessness in order to promote youth informed decision making

When asked about ways to support YEH interested in learning more about or receiving the COVID-19 vaccine, youth advocated for strategies that could improve vaccine messaging and outreach. These youth-driven opportunities to promote COVID-19 vaccine confidence and access included: 1. promoting autonomy and agency, 2. utilizing trusted sources of information, and 3. improving vaccine access (Fig. 2).

### Emphasizing autonomy

Youth reported a desire to have the ability to choose if they receive the COVID-19 vaccine, when and where to get vaccinated, and which type of vaccine to receive. When expressing their hope to be informed about their options and to decide their vaccination status on their own volition, one youth reported: *“They just want to have the options. It shouldn't be – It shouldn't feel like it's being pushed.”* Many youth commented on their hope to have autonomy in their vaccine decisions, but also to respect the vaccine decisions of others around them. For example, one youth commented, *“I gotta see how it’s gonna play out and stuff like that and I didn’t really give my opinion because as time went on, everyone thought of it as political or other things, and I just thought that it’s like a regular flu shot. You can either get it or not get it, it’s your decision.”*

### Leveraging trusted sources of information

#### Using trusted spaces to display vaccine information

Youth and staff identified spaces effective at conveying COVID-19 vaccine information and outlined strategies to promote health and vaccine literacy in these spaces. YEH commented that trusted vaccine information should appear on mass transit, a space where agency information is frequently displayed: *“You guys are on the buses. That's why I was like put it by all the [agency] advertisements.”* Youth identified several other spaces, as well: *“Having information in frequently visited locations such as tobacco stores, liquor stores, the libraries.”*

The people conveying COVID-19 vaccine messaging were important to YEH, coupled with familiarity and trust within YEH-serving agencies. One staff member discussed the importance of involving YEH-serving agency staff members in this process. *“I think sometimes staff who already have a relationship with young people can be the right messenger. And, yeah, it's hard, but again I feel like our space is one of the only spaces maybe where they are going to have access to solid information, and we are someone they trust.”*

#### Including youth in developing health-related messaging

When we showed YEH existing COVID-19 vaccine messaging from external sources, they broadly stated the messaging did not resonate with them. When asked their opinions on these videos, youth imagined different ways to present this information to youth: *“It would have been better if they had, like, 50 people that were vaccinated and 50 people that were unvaccinated go through these questions…Cause all we looking at right now is statistics and their data that they're providing us.”* Youth also stated the importance of diversity in the people whose experiences were being presented: “*I feel like they should have had teens and elderly people and young adults. Like, they can say what their different side effects were or something like that, or how it worked for them. And people with different races.*” One staff member identified YEH as being the best source of vaccine information for other YEH. *“Getting other young people to join with other scientists or doctors and those young people maybe being trained in and educated about the vaccine…because you have to have that relatable aspect there…”.*

#### Representation and trusted COVID-19 information sources

When YEH were asked about strategies to disseminate information to other YEH, one youth suggested that a phone line staffed by YEH would better equip YEH to make informed vaccination decisions. *“I say a youth line. A youth line answered by youth.”*

YEH and staff discussed the existing lack of representation in health information messengers and the importance of messaging from people with shared experiences and identities, including racial identity. A staff member noted:

*“Well, for sure I think for our youth, it’s really important to see people who look like them sharing the message. Because, maybe this is really blunt, but if you see a bunch of old white doctors talking about it, youth are gonna be like, why should I listen to them? Versus seeing people who are really representational and experts in the field.”*

This lack of representation presented concerns about racial representation in vaccine development; for example, one youth mentioned *"lack of testing—like the subjects in the trials mostly being white. How can we be sure it’s not gonna affect different people of color differently? And lack of POC doctors, as well."*

A staff member discussed the importance of education in trusted spaces and the burden that falls upon healthcare providers and public health messengers in tackling misinformation.

*“It's really the education component. Our youth are not really getting consistent information and it's not the right information a lot of the times. So a lot of the barriers are really trying to push out all of the false information that they have to get them to a point of comprehension and comprehending that this is actually going to benefit you and actually going to help you and the people around you.”*

### Improving vaccine accessibility

By addressing structural barriers, like insurance and transportation, and addressing concerns about managing short-term vaccine side effects, YEH in this study suggested creative, youth-centered strategies to improve vaccine confidence and uptake among YEH. They suggested developing improved vaccine access by addressing structural barriers, which included being clear about not requiring insurance or personal documentation in the future, offering transportation to vaccine sites or bringing the vaccine sites to places where youth already interact, and offering vaccine aftercare for young people. One youth suggested,*“I think that if they made the vaccine completely free and not insurance-based or anything else like that, or they had COVID-related vehicles that went to people, such as elderly or youth, or stuff like that. I think that would be a real game changer.”* Another youth suggested, *“Just COVID information stations and COVID vaccine stations.”*; when asked to clarify, they specified, *“…You have a section where you can go and read all your information about it and then choose whether you want to stay and get the vaccine or if you want to take some time to think on it.”*

Post-vaccine aftercare was also suggested by several youth: *“Help them, if they have adverse reactions, like a fever or something the next day, be able to make sure that they can access medication to control the fever, if they'd want to.”* A different youth specifically recalled, *“When I went to get mine, CVS let me use their chair. They had a designated chair where you had to sit for a period of time after you got your COVID shot. I didn’t really think much of it at the time, but it was helpful.”*

## Discussion

This qualitative study of YEH’s perspectives on COVID-19 vaccination provides insight into key factors influencing YEH’s COVID-19 vaccine confidence and suggests several opportunities for enhancing confidence and access. Though data on the topic of COVID-19 vaccine confidence among YEH is limited, this builds upon several recent studies in Toronto: one explored the impact of COVID-19 on 2SLGBTQ YEH, which identified the need for healthcare that is both affirming and addresses social and health issues among this population [27]. Our study focuses on youth experiencing homelessness more broadly but similarly elucidates themes of mistrust and information accessibility among our most salient findings, as well as fear of vaccine side effects as an additional decision influencer.

Our study builds on prior work suggesting similar barriers to vaccine confidence. One study conducted in Toronto described COVID-19 vaccine attitudes among 2SLGBTQ YEH found vaccine hesitancy in this population to be related to mistrust in the healthcare system, lack of targeted public health information about vaccines, safety and side effect worries, and lack of accessibility [16]. The U.S. government and healthcare systems have a long-standing history of oppressing marginalized communities, particularly communities of color, which has enduring effects. As highlighted in our study, young people are very knowledgeable and acutely aware of the structural inequities and systemic oppression that exist within systems, how these inequities disproportionately affect their own communities, and how the government has been involved in perpetuating these. We found that youth frequently commented on historical harms in U.S. healthcare that unethically and explicitly harmed people of color, such as references to forced sterilization programs and to the Tuskegee Syphilis study, which often manifested for them as present-day mistrust of systems and, therefore, mistrust of the COVID-19 vaccine. Decades of research find that healthcare barriers for YEH relate to mistrust of authoritative systems and perceptions of discrimination [16, 28, 29]. Likewise, YEH may be disconnected from healthcare at baseline, due to compounding healthcare inequities, and it is likely that access to care was further limited by the pandemic [4, 13, 30].

Youth in general are vulnerable to false information, especially during the COVID-19 pandemic, through the use of social media. Some investigations have found that people who were younger and of lower income or education status were more likely to adopt COVID-19-related conspiracy beliefs [31]. It is well-documented that youth can be harmed by exposure to mis/disinformation and may unknowingly circulate this misinformation among their peers; however, education can help young people develop critical thinking skills to learn to decipher which information is reliable [32]. While social media has perpetuated false information, it can also be an opportunity to serve as a source of sharing up-to-date, reliable science [33], particularly as the youth in our study hoped to be involved and represented in youth-friendly vaccine messaging.

Our study underscores the importance of centering the diverse voices and lived experiences of youth in future public health pandemic planning and vaccination outreach to develop youth-centered, culturally-specific vaccination and public health messaging. Such strategies could include developing or bolstering youth advisory boards, offering financial incentives to youth for their participation and expertise, or creating space to invite youth to be involved in decision making at the local, state, or federal policy level.

In promoting vaccine confidence among unstably housed youth, a multi-layered and interdisciplinary approach could help address the historical harms, marginalization, and unique barriers, needs, and strengths of this population. This approach allows youth to make informed decisions about their body and could promote autonomy in vaccination decision making. Collaborative, cross-sector approaches can be implemented to improve and streamline critical health and social services for YEH [4]. In conjunction with an interdisciplinary approach, educational interventions can be beneficial in improving vaccine uptake, as highlighted in studies suggesting improved HPV vaccine acceptance as a result of educational interventions among adolescents and young adults [34, 35]. Our findings emphasize young people’s interest in improved COVID-19 education by utilizing reliable and trusted sources of information.

Health education directed towards YEH, including messaging from public health sources, social media, healthcare providers, and other trusted channels of information, should address concerns and barriers to vaccination in ways that encourage autonomy while facilitating healthcare access. Several studies have identified immigration status as a barrier to both vaccination and healthcare utilization. To address this, we recommend targeted public health initiatives focused on facilitating vaccine access among people undocumented immigrant populations [36, 37]. Similarly, federal policy restricting access to healthcare without appropriate identification is a limitation or perceived limitation to utilizing healthcare or vaccination; advocacy at the national policy level can be prioritized to ensure equitable, barrier-free access to healthcare [36]. Through education and increased access to tools identified to promote vaccine uptake, healthcare providers and public health professionals can improve healthcare access among YEH.

Importantly, our study emphasizes the value of centering the voices of marginalized youth, specifically YEH, to uplift youth perspectives and promote equitable uptake of the COVID-19 vaccine among YEH. Our findings identify youth-centered, anti-oppressive opportunities for improved vaccine messaging that could ultimately help reduce adverse health outcomes due to COVID-19 among YEH. A strength of our study includes centering the voices of community partners, including youth themselves and youth-serving staff. Uplifting youth voice was an integral piece of this study, occurring throughout community partnership building, interview design, coding methodology, and iterative community feedback. By creating safe spaces for YEH to share their lived experiences and inviting them to participate in advancing public health approaches, youth were empowered in their own health decisions and as leaders in the public health landscape that impacts both them and their peers.

Though qualitative data are not intended to be representative, one limitation of this study is generalizability, given that the subset of YEH who were engaged were already connected with YEH-serving agencies in an urban county. Therefore, the perspectives of YEH who were not connected to housing agencies, for example those who were staying in encampments or staying in cars, were not elicited. Similarly, youth in our study were primarily recruited in a large metropolitan area; our findings may differ from the perspectives of YEH in rural settings. Although we did collect youth demographic data through an optional survey, several youth declined the survey. Given that our demographic data is skewed, more complete demographic information may have provided different context to the data. Our study was cross-sectional in nature, with focus groups and key informant interviews conducted between October and November of 2021. Perspectives may have evolved as COVID-19 vaccine access and public health measures change over time.

In conclusion, our findings highlight important considerations when working to promote COVID-19 vaccine confidence and uptake among YEH and pave the way for implementation and evaluation of the opportunities identified by youth about COVID-19 vaccine messaging, as well as other vaccines, recognizing the complex factors that informed youth decisions regarding vaccinations. As part of the larger community- and youth-engaged project in which this study took place, we identified and implemented several of these approaches, in collaboration with youth and YEH-serving agencies, including youth-friendly, culturally-responsive messaging, site-based health events, vaccine aftercare kits, and staff training resources [38]. These interventions reinforced the importance of collective learning as we continued to navigate the COVID-19 pandemic. In future work, it will be essential to further describe the perspectives of YEH who are not connected with YEH-serving agencies and of youth in rural areas to reflect a broader and more diverse group of youth.

## Data Availability

The datasets used and/or analyzed during the current study available from the corresponding author on reasonable request.

## Abbreviations

YEH: Youth Experiencing Homelessness
PEH: People Experiencing Homelessness
